# Cavity optomechanics mediated by a quantum two-level system

**DOI:** 10.1038/ncomms7981

**Published:** 2015-04-27

**Authors:** J.-M. Pirkkalainen, S.U. Cho, F. Massel, J. Tuorila, T.T. Heikkilä, P.J. Hakonen, M.A. Sillanpää

**Affiliations:** 1 Department of Applied Physics, Aalto University, PO Box 11100, FI-00076 Aalto, Finland; 2 Department of Applied Physics, Low Temperature Laboratory, Aalto University, PO Box 15100, FI-00076 Aalto, Finland; 3 University of Jyväskylä, Department of Physics, Nanoscience Center, University of Jyväskylä, PO Box 35 (YFL) FI-40014 University of Jyväskylä, Jyväskylä, Finland; 4 Department of Physics, University of Oulu, FI-90014 Oulu, Finland

## Abstract

Coupling electromagnetic waves in a cavity and mechanical vibrations via the radiation pressure of photons is a promising platform for investigations of quantum–mechanical properties of motion. A drawback is that the effect of one photon tends to be tiny, and hence one of the pressing challenges is to substantially increase the interaction strength. A novel scenario is to introduce into the setup a quantum two-level system (qubit), which, besides strengthening the coupling, allows for rich physics via strongly enhanced nonlinearities. Here we present a design of cavity optomechanics in the microwave frequency regime involving a Josephson junction qubit. We demonstrate boosting of the radiation–pressure interaction by six orders of magnitude, allowing to approach the strong coupling regime. We observe nonlinear phenomena at single-photon energies, such as an enhanced damping attributed to the qubit. This work opens up nonlinear cavity optomechanics as a plausible tool for the study of quantum properties of motion.

## Abstract (web summary)

Radiation pressure can control the motion of a nanoscale resonator, but pushing this to the quantum limit is difficult because the influence of a single photon is tiny. Here, the authors boost the radiation–pressure interaction by six orders of magnitude using a Josephson junction qubit

## Graphical abstract

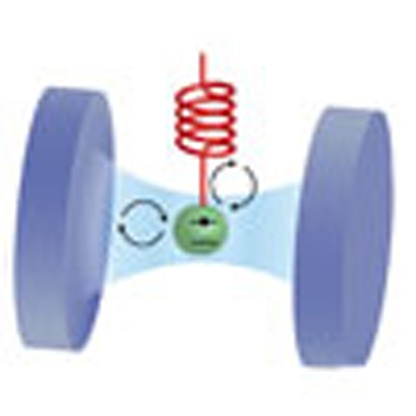

The basic setup of cavity optomechanics^1^,^2^ involves an otherwise ordinary optical Fabry–Perot cavity resonator, but having one of the mirrors compliant for mechanical vibrations. The oscillatory displacement *x* of the mirror couples to the length that corresponds to the frequency *ω*_c_ of the cavity. This interaction can also be interpreted as that resulting from the radiation pressure caused by the cavity photons and characterized by the coupling energy *g*_0_≡(∂*ω*_c_/∂*x*)*x*_zp_. Here, *x*_zp_ is the zero-point motion amplitude. Cavity optomechanics has recently received a lot of attention, for it has turned out as an excellent test system for investigations on quantum–mechanical properties of the motion of nearly macroscopic bodies^3^,^4^.

Several different physical systems have been demonstrated, which realize the radiation-pressure interaction between electromagnetic and mechanical modes. In particular, the frequency of the electromagnetic cavity need not be optical. One of the most successful realizations in the recent past has been the use of microwave-regime superconducting cavities made with lithographic techniques^3^,^5^,^6^. Here, the displacement changes the effective capacitance *C*(*x*) of the cavity and hence its resonance frequency *ω*_c_, and the coupling energy is *g*_0_=(*ω*_c_/2*C*)(∂*C*/∂*x*)*x*_zp_.

In all the physical realizations, except in the case of microscopic motional degrees of freedom^7^,^8^, the radiation-pressure coupling energy is much smaller than the cavity decay rate *κ*. Therefore, the cavity has to be strongly irradiated up to a high photon number *n*_c_≫1 in order to enhance the intrinsically small interaction. This, however, linearizes the interaction and creates two linearly coupled harmonic oscillators, which is a somewhat limited system. Hence, one nowadays strives to enhance the magnitude of the interaction to exploit the intrinsic nonlinearities^9^,^10^,^11^,^12^,^13^,^14^. The next goal is to reach a strong coupling threshold *g*_0_≳*κ* where some quantum phenomena become observable^9^,^11^,^13^,^15^,^16^. The largest radiation-pressure coupling values *g*_0_/2*π*≃1 MHz have been demonstrated in the optical frequency domain (ref. 4). These systems also possess the largest ratio of the coupling versus cavity decay *κ*, which is, *g*_0_/*κ*∼0.2%. This is still two and half orders of magnitude away from strong coupling.

Here we build on the scheme proposed in ref. 17. The scheme is motivated by several successful experiments coupling superconducting Josephson junction quantum circuits to micromechanical resonators^18^,^19^,^20^,^21^,^22^, although canonical radiation–pressure phenomena have not been observed in those setups. We demonstrate a microwave-regime optomechanical device that allows for high values of *g*_0_/*κ*, and at the same time, displays phenomena not previously observed in cavity optomechanics either in the microwave or the optical frequency range.

## Results

### The basic idea

**Figure 1:**
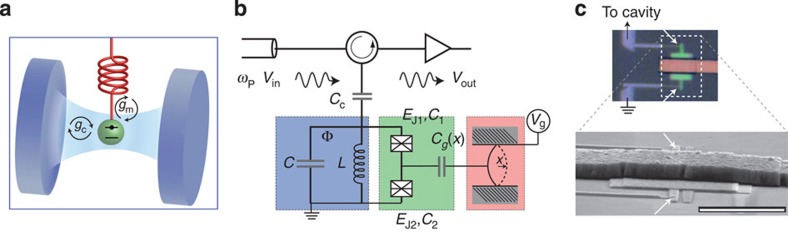
Optomechanics with a qubit. (**a**) Illustration showing the idea for introducing a two-level system (qubit or artificial atom) into a cavity optomechanical setup. Inside the cavity (blue) there is a quantum two-level system (green), which is mechanically compliant (red). (**b**) Equivalent electrical circuit of the microwave design with the same colour codes as in **a**. The parameters in the equivalent circuit are *C*≃0.33 pF, *L*≃3.2 nH, *C*_g_≃1.8 fF, *C*_Σ_≃4.9 fF, *E*_J_=*E*_J1_+*E*_J2_≃10.5 GHz, *E*_C_≃5.3 GHz. The reflected pump microwave *V*_out_ is detected outside the cryostat. The Josephson junctions forming the qubit are marked by crossed boxes, and the mechanical resonator is coupled to the qubit island via a movable capacitance *C*_g_(*x*). (**c**) Micrographs of the device showing the qubit and the mechanical resonator. The Josephson junctions are marked by arrows. Scale bar, 2 μm.

The device consists of a tripartite system made with patterned thin films of superconducting aluminium on a silicon chip, containing first of all a microwave resonator (called cavity in the following). Another part is a micromechanical resonator and the third is a charge quantum two-level system (qubit), which consists of small Josephson tunnel junctions and mediates the interaction between the former two parts (see Fig. 1 and Supplementary Fig. 1). The bare cavity and the mechanical modes have the frequencies 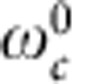 and 
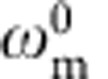
, and their dynamical variables are the creation and annihilation operators *a*^†^, *a* and *b*^†^, *b*, respectively. The qubit is represented by the Hamiltonian *H*_QB_=−*B*_*x*_*σ*_*x*_/2−*B*_*y*_*σ*_*y*_/2−*B*_*z*_*σ*_*z*_/2. Here, *σ*_*j*_ are Pauli matrices and *B*_*j*_ are the controllable pseudomagnetic fields, which also depend on the cavity and mechanical coordinates. The level spacing of the qubit is 
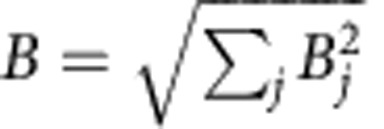
. More discussion of the Hamiltonian is found in Supplementary Methods.

A fundamental reason to expect rich optomechanical physics in the scheme owes to the nonlinearity of the quantum two-level system^17^,^18^,^19^,^20^,^21^,^22^,^23^,^24^,^25^. An important ingredient here is the large d.c. voltage bias *V*_g_ applied to the mechanical resonator represented as a movable capacitance *C*_g_(*x*). If coupled to a two-level system (qubit), this creates a longitudinal coupling *g*_m_(*b*^†^+*b*)*σ*_*z*_ in the natural basis of the charge qubit, with a maximal effect on the energy of the qubit by the mechanical resonator. When coupling this qubit to a cavity with the transverse interaction *g*_c_(*a*^†^+*a*)*σ*_*x*_, the cavity frequency will experience a Stark shift 
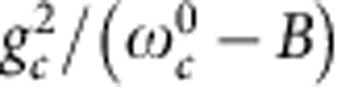
. This Stark shift contains a contribution from the vibrations due to their effect on qubit level spacing. The system then closely approximates the radiation-pressure interaction between the effective cavity mode (frequency *ω*_c_) and the effective mechanical mode (frequency *ω*_m_), which both are Stark shifted from the bare frequencies. The radiation-pressure coupling strength between these modes can be amplified in this setting by a factor of the order of *CV*_g_/(2*e*), which amounts to ∼4 to 6 orders of magnitude for typical experimental parameters (ref. 26). Moreover, there are intriguing nonlinear corrections to the basic setup, first of all, a cross-Kerr type interaction in the next higher order. Most importantly, the damping of the mechanics becomes affected by the nonlinearities.

The physical device (see Fig. 1c and Supplementary Fig. 2) is fabricated by standard electron-beam lithography. The cavity is a 5-mm long superconducting microstrip resonating at the (bare) cavity frequency 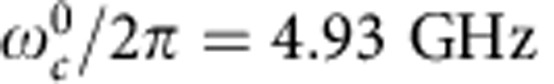. The charge qubit has the island capacitance *C*_Σ_=*C*_g_+*C*_1_+*C*_2_, which gives the charging energy of a single electron *E*_*C*_=*e*^2^/2*C*_Σ_. It is of the same order as the Josephson energy *E*_J_, which is *E*_J_/*E*_C_≃2.0. A strong qubit-cavity coupling with the coupling energy up to ∼500 MHz (depending on the qubit bias) sets the system near the ultrastrong coupling^27^ of circuit QED, and hence gives rise to a pronounced Stark shift of the cavity. The micromechanical resonator is made as a 2-μm wide bridge suspended across a 50 nm vacuum gap atop the qubit island (ref. 21) and resonating at the frequency *ω*_m_/2*π*≃65 MHz of the lowest flexural mode. The qubit-mechanics coupling varies here between *g*_m_/2*π*≃80 MHz…160 MHz depending on the value of *V*_g_. We note that in spite of the large coupling exceeding the bare mechanical frequency, the Schrieffer–Wolff approach of deriving the radiation-pressure interaction is justified (see Supplementary Methods).

### Measurements

**Figure 2:**
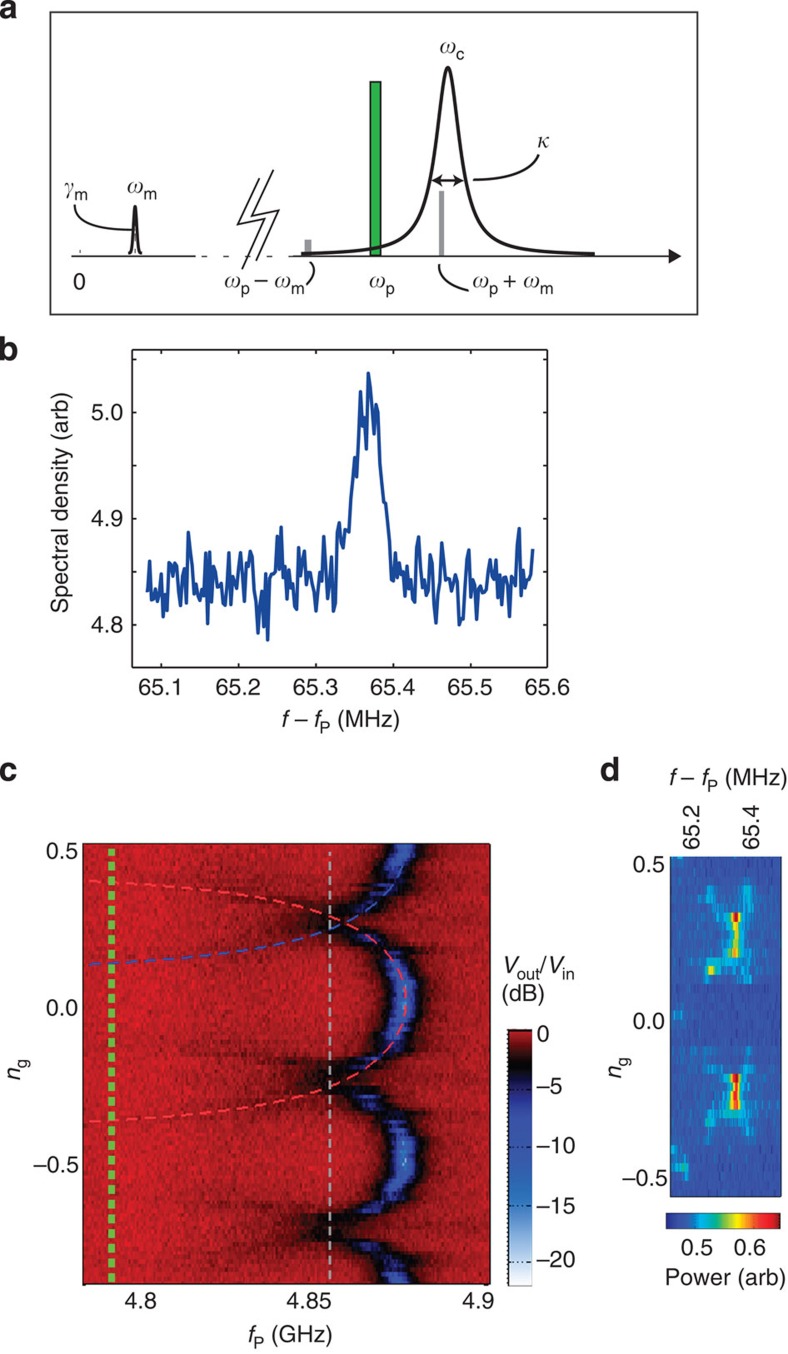
Basic characterization. (**a**) The frequencies involved: the vertical axis gives the spectrum of either the cavity (*ω*_c_) or the mechanical resonator (*ω*_m_). The pump (frequency *ω*_p_) is applied around the detuning Δ≃−1 below the cavity frequency. (**b**) Thermal motion measured at 20 mK. The parameters are Δ≃−1, *n*_c_≃0.05, *n*_g_=0.25, *V*_g_=6.5 V. (**c**) Gate charge modulation of the cavity resonance absorption. The resonance *ω*_c_(*n*_g_) of the effective cavity appears as the periodic black-blue line. The dashed red and blue lines are theoretical fits to two of the branches, obtained by using the qubit-cavity Hamiltonian^17^ in numerical diagonalization. The flux bias was Φ/Φ_0_≃0.39. The vertical green and grey lines represent the frequencies *ω*_p_ and the sideband *ω*_p_+*ω*_m_, respectively, used to obtain the data in Fig. 3a,b. (**d**) Spectral density of the emission from the cavity around the mechanical sideband.

In all the measurements of the mechanical motion we use the basic idea of cavity optomechanics shown in Fig. 2a, where the cavity is irradiated by the pump microwave at a frequency *ω*_p_, which usually resides near the red sideband *ω*_c_−*ω*_m_. The pump detuning Δ≡(*ω*_p_−*ω*_c_)/*ω*_m_ is hence around Δ∼−1. The motional sidebands at ±*ω*_m_ about the pump give information about the mechanics. In the following, the sideband closer to the cavity is studied. The main result of the radiation-pressure interaction is the ‘optical spring effect' which modifies both the mechanical frequency and damping. The changes exerted are denoted as 
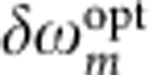
, and 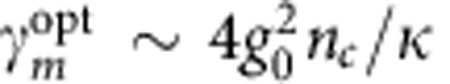, respectively.

The experiments are performed in a dilution refrigerator at 20 mK temperature. We first identify the mechanical resonance by pumping the cavity very strongly up to *n*_c_∼10^9^ such that the Josephson effect, which has its influence only in the low-photon regime *n*_c_∼1, becomes insignificant, and what is left behind is a linear cavity with the bare frequency 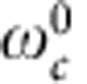. The estimated bare optomechanical coupling between the bare cavity and the mechanics is only *g*_0_/2*π*∼1 Hz. Such a small coupling does not yet allow for observing the small thermal motion of the mechanical resonator at a low temperature. We hence actuate the motion via the gate line, up to about 10-pm displacement amplitude, obtaining the resonance curve shown in Supplementary Fig. 3. This way we determine the intrinsic mechanical frequency *ω*_m_/2*π*≃65 MHz and linewidth that is *γ*_m_/2*π*=15 kHz. Notice that *ω*_m_ decreases when *V*_g_ deviates from zero due to the well-known electrostatic softening of the mechanical spring.

Next, we decrease the pump power by about 10 orders of magnitude down to *P*_p_∼1 fW. Although power levels are drastically reduced, the detection sensitivity is enhanced and we can observe the sideband peak corresponding to the thermal motion, shown in Fig. 2b, at 20 mK temperature, amounting to about six phonons at equilibrium. The peak frequency is somewhat shifted from the calibration experiment, and the linewidth of this peak is about twice the intrinsic linewidth. As discussed below, this results from the qubit-induced nonlinearities. We now turn into careful characterization of the novel type of optomechanical interaction. We first show that the device displays the basic physics due to the radiation-pressure interaction, namely, the optical spring effect. As discussed below, the latter causes the mechanical frequency and damping to differ from the intrinsic values.

We now characterize the basic behaviour of the microwave cavity. As seen in Fig. 2c, the frequency of the effective cavity depends periodically on the gate charge *n*_g_=*C*_g_*V*_g_/2*e*. Each period corresponds to the tunnelling of one extra Cooper pair onto the qubit island. Here we have a large dc voltage *V*_g_∼5…10 V, which is needed to establish the electromechanical interaction, hence *n*_g_>10^4^. However, because of periodicity, we henceforth refer to *n*_g_ via its non-integer part for simplicity. On top of the large d.c. voltage, we use a fine tuning voltage to adjust the radiation-pressure physics by tuning the gate charge within one period. The period of the cavity response is one electron (0.5) instead of two as expected. This is due to a quasiparticle slowly jumping on and off the island^21^,^28^. We thus see a double image, shifted by one electron, of the cavity transition. These are marked by the red and blue dashed lines in Fig. 2c. At a given *n*_g_, there are hence two different cavity frequencies. The system switches between these states while the quasiparticle randomly tunnels on and off the island at the typical rates well below a MHz (refs 29, 30). These rates are much slower than the mechanical frequency, and hence in the long-time average we see responses from both cavity states superimposed on top of each other.

From the geometry we find ∂*C*_g_/∂*x*≃24 nF/m and *x*_zp_≃6 fm. We determine the coupling from the experimental data using *g*_0_=(∂*ω*_c_/∂*x*)*x*_zp_, where (∂*ω*_c_/∂*x*)=*V*_g_/2*e*(∂*ω*_c_/∂*n*_g_)(∂*C*_g_/∂*x*) can be related to the slope of the gate charge modulation of the cavity frequency seen in Fig. 2c. For *n*_g_=0.25 where the cavity is reasonably well visible, we have (∂*ω*_c_/∂*n*_g_)≃(2*π*)·250 MHz and hence *g*_0_/2*π*≃0.5 MHz when *V*_g_=4.6 V. Since |*g*_0_(*n*_g_=0.25)|=|*g*_0_(*n*_g_=0.75)|, this value is the same for both cavity branches.

### Gate charge dependence

One can characterize the radiation-pressure physics as a function of, for example, the value of the gate charge *n*_g_. Changes in *n*_g_ change the value of *g*_0_, which is proportional to the slope (∂*ω*_c_/∂*n*_g_). However, a change in *n*_g_ has also other consequences. With a given pump frequency, the pump detuning Δ changes according to the effective cavity frequency. Similarly, also the effective mechanical frequency *ω*_m_ will change (due to Stark shift) with *n*_g_. Finally, these effective modes exhibit the radiation-pressure interaction visible as further changes in frequency and damping due to the optical spring effect. We denote the resulting total frequency of the mechanics as 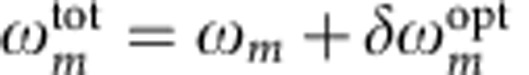. The total damping is given by 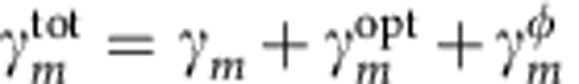, where the last term 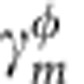 is due to the qubit and is discussed below. In Fig. 2d we show a result of this kind of a measurement, allowing us to connect the thermal emission peak with the cavity response. A maximum emission occurs at *n*_g_≃0.25, which here coincides with the sideband resonance and hence the expected maximum signal. The two crossing branches are associated with the two cavity branches.

**Figure 3:**
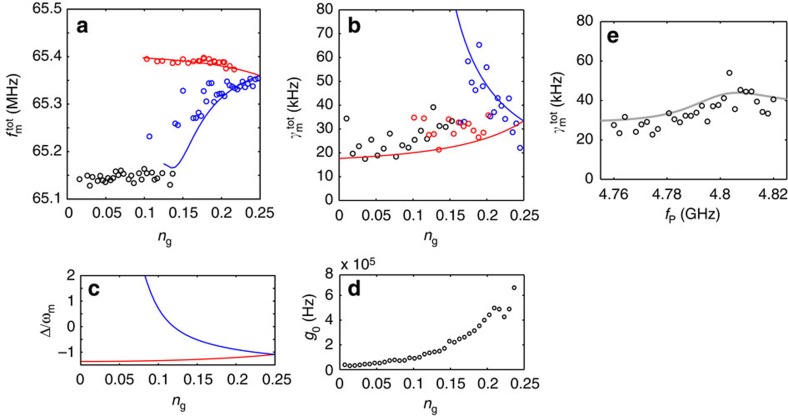
Optomechanics enhanced by a qubit. (**a,b**) Optical spring effect as a function of gate charge. The solid lines are a fit to theory and are plotted in the region where detuning and coupling are such that the peaks are visible. The red and blue colours refer to the two cavity branches as in Fig. 2. The black circles present data that presumably originate from higher excited levels of the qubit (see main text). The pump frequency *ω*_p_/2*π*=4.79 GHz, and the sideband (*ω*_p_+*ω*_m_)/2*π*=4.855 GHz. (**c**) Illustration of the pump detuning for the two branches for the measurements in **a** and **b**. (**d**) Radiation-pressure coupling extracted from *ω*_c_(*n*_g_). (**e**) Optical spring as a function of pump detuning, at fixed gate charge *n*_g_=0.25. The solid line is a fit to theory. In all data, the cavity photon number is in the range 0.1...1. This is estimated based on the input power and cable attenuation, and in the end fine-tuned as an adjustable parameter. In all the panels, *V*_g_=4.6 V.

We next look in detail at the frequency and linewidth of the thermal motion peak in the measurement as in Fig. 2d. The extracted parameters are displayed in Fig. 3a,b revealing three distinct sets of peaks. To understand this data, we first calculate the Stark-shifted *ω*_m_ and *ω*_c_, as well as *g*_0_ from the diagonalization of the qubit-cavity Hamiltonian (see Supplementary Methods) and then apply the results in refs 17, 31 to obtain a prediction for the optical spring. Each of the two cavity branches causes its own sideband response. These arise via the detuning and coupling shown in Fig. 3c,d. The blue curves in reality originate from *n*_g_ values between 0.25...0.5, but reflect to the left about *n*_g_=0.25 due to the quasiparticle. The data and theory are marked with the compatible red and blue colour codes in Figs 2 and 3 and Supplementary Fig. 4. We obtain a good agreement between the experiment and theory for the mentioned two branches. However, the peaks visible around *n*_g_=0...0.15, marked in black, are not directly explained by this model. We attribute them to that the qubit spends some part of the time in the excited state as observed in this kind of measurements^21^. The excited state gives rise to another cavity frequency above *ω*_c_, having the highest ∂*ω*_c_/∂*n*_g_ around *n*_g_=0. The qubit being excited, however, is beyond the current model and we cannot make a quantitative comparison to theory.

### Damping due to the qubit

In the rest of the paper, we select a fixed value *n*_g_=0.25, which simplifies the analysis since there is only one cavity branch and a constant *g*_0_. Another way to examine the radiation-pressure interaction is to change the pump detuning (Fig. 3e). The total damping is maximized close to the sideband resonance in agreement with theory.

**Figure 4:**
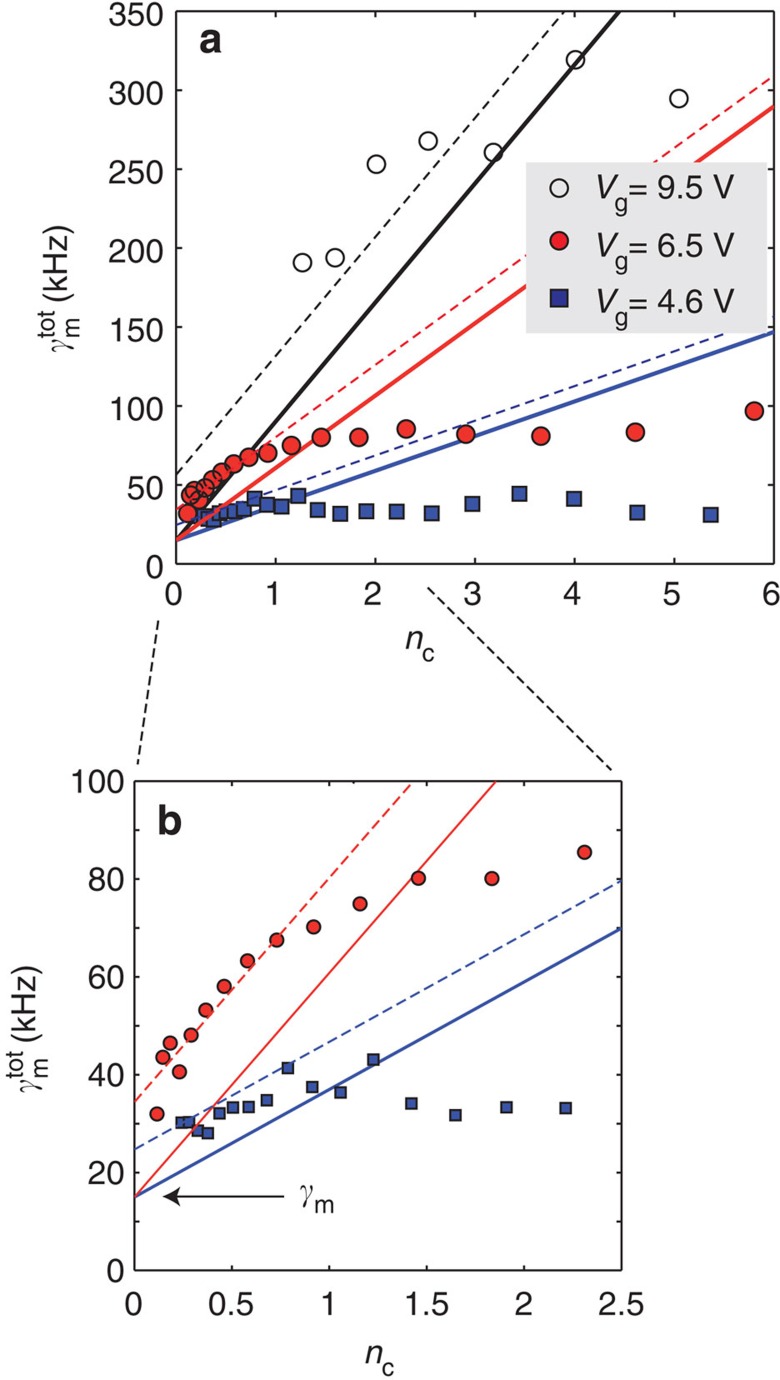
Nonlinear cavity damping. (**a**) The total (tot) mechanical linewidth as a function of cavity photon number. Red circles: *V*_g_=6.5 V, *g*_0_/2*π*≃0.7 MHz. Black circles: *V*_g_=9.5 V yielding higher *g*_0_/2*π*≃1.0 MHz. Squares: smaller *g*_0_/2*π*≃0.5 MHz obtained with *V*_g_=4.6 V. The solid lines are expectations from the basic linear model, that is 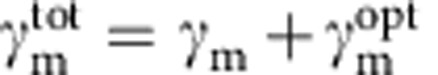. The dashed lines include the qubit-induced extra linewidth, 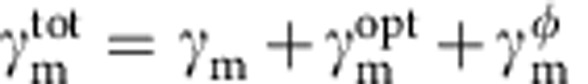. (**b**) Zoom-in of the linear regime of **a**. In all the data, Δ≃−1, Φ/Φ_0_≃0.39.

The total damping should increase linearly with the pump photon number *n*_c_ owing to the contribution 
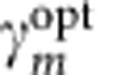
, moreover, it should depend quadratically on *g*_0_ at a given *n*_c_. We test this in Fig. 4 by varying the photon number at a few fixed values of *g*_0_, which are set by changing the gate voltage *V*_g_. The data points are extracted from the data sets in Supplementary Fig. 5, which we here show as an example of thermal motion peaks. As observed in Fig. 4, the damping towards *n*_c_→0 is clearly higher than the intrinsic *γ*_m_. The extra damping is attributed to that the qubit opens another dissipation channel due to the hybridization of the qubit and the mechanics^17^, analogously to the Stark shift, and is given by


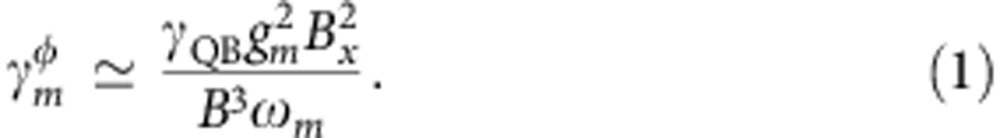


Here, *γ*_QB_ is the qubit relaxation rate. We could not independently measure it, but a good fit of the data is obtained with a reasonable value *γ*_QB_≃(2*π*)·60 MHz.

Although the damping due to the basic cavity cooling 
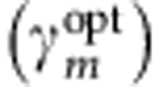
 gives rise to back-action sideband cooling of the mechanical resonator, further theoretical work is needed to understand the effect of the qubit contribution 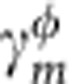 as well as the nonlinearities such as the cross-Kerr effect on the final temperature.

In Fig. 4 we see that the damping grows first linearly up to about *n*_c_∼1 and then saturates. The saturation of the radiation-pressure physics around *n*_c_∼1 is due to reaching the limits of the linear regime of the effective cavity. This is set by the requirement that the total phase excursions 
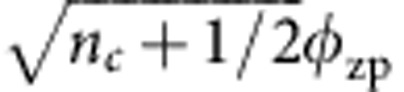
 should be small as compared to 2*π* in order not to hit the nonlinearity of the Josephson cosine function. This is analogous to the transition of a trapped ion out from the Lamb–Dicke regime (see Supplementary Methods). Here, the phase zero-point fluctuation is *φ*_zp_∼0.2, which agrees with the scale we observed.

## Discussion

The largest *g*_0_/2*π*≃1.6 MHz obtained in this work is achieved by half of a flux quantum flux bias, and setting a high gate voltage *V*_g_=9.5 V. This value is the largest ever reported in a cavity optomechanical setting. This was achieved by integrating, for the first time, a quantum two-level system into such a setup, allowing boosting of the radiation-pressure coupling by six orders of magnitude. The ratio of coupling to the cavity linewidth, ∼4%, can likely be further increased by designing the coherence properties of the qubit. We estimate that boosting factors up to eight orders of magnitude, giving a radiation-pressure coupling up to 100 MHz, are in principle possible with an optimized device (see Supplementary Fig. 6). The setup introduces long-sought nonlinearities into the framework of cavity optomechanics and enables their utilization, for example, in Fock-state measurements of the phonon states, or for tests of the foundations of quantum mechanics^32^,^33^,^34^.

## Methods

### Chip layout

A photograph of the chip is shown in Supplementary Fig. 2. The ends of the meandering inductance are close to each other such that the double-junction charge qubit can be placed to connect the ends of the meander, which is required for the scheme. The device is fabricated on a regular silicon substrate using a similar process as in ref. 21. The device was designed to not have a too small cavity capacitance for the system to reside in the Lamb–Dicke limit for qubit-cavity coupling (see discussion in Supplementary Methods). Most of the capacitance is provided by a bonding pad-like structure visible between the coupling capacitance and the meandering inductance, marked *C* in Supplementary Fig. 2.

### Experimental setup

The measurements are done at the base temperature of a dilution refrigerator using the cabling setup as shown in Supplementary Fig. 7. The pump microwave reflects from the cavity and is amplified at a 4 K stage using a low-noise amplifier. The system noise temperature is in the range of 3...4 K.

Filtering the room temperature noise away from reaching the sample via the cable connected to the mechanical resonator (that is, qubit gate) is challenging because the typical solution (resistive attenuators) cannot be used owing to the high voltages involved. We hence use reflective filtering. From the qubit point of view as well, the filter needs to be reactive, because dissipative 50 Ω environment could cause too high spontaneous decay. The filtering task is carried out by a combined bias-T and low-pass filter, marked yellow in Supplementary Fig. 7. Without the filter, we observe substantial excitation of the qubit to the higher levels, such that the spectrum of the effective cavity changes qualitatively and cannot be modelled by ground-state transitions. With the filter installed, the excitation is modest, and cavity response well understood. The filter provides enough transmission (−33 dB) at frequencies around *ω*_m_ such that we can actuate the mechanics if needed.

### Cavity characterization

The cavity frequency has a strong dependence on the magnetic flux Φ applied through the loop, as shown in Supplementary Fig. 8, which displays the microwave reflection response quite similar to Fig. 2c in the main text. The cavity resonance appears as the yellow-blue coloured dip varying between 4.99 and 4.84 GHz, periodically in flux with the period of one flux quantum Φ_0_. In the measurement, we applied noise to the gate such that the response is a weighted average over all the possible gate *n*_g_ values. In the range ∼0.4…*n*_g_…0.6 the resonance is not well visible because of the strong gate dispersion. The theoretical curves plotted on top of the data in Supplementary Fig. 8 are obtained by numerical diagonalization of Supplementary Equation 9, but disregarding the mechanics (that is, *n*_g_(*x*)→*n*_g_), which here is insignificant. The overlaid curves indicate a good match between the experiment and the modelling.

The cavity linewidth *κ* has a strong dependence on the qubit gate charge, as displayed in Supplementary Fig. 9. While the changes are due to the qubit, we cannot make a definite conclusion of whether it is caused by qubit relaxation or dephasing. We also checked that simulating the inhomogenous broadening by establishing the theory with bare *κ*, and selecting a ensemble of *f*_c_, and averaging over the ensemble does not markedly change the results.

## Author contributions

J.-M.P. and S.U.C. designed and fabricated the devices. J.-M.P. carried out the measurements and a part of the data analysis. F.M. and J.T. and T.T.H. provided the theoretical results. M.A.S. conceived the work and designed the experimental setup with P.J.H.

## Additional information

**How to cite this article:** Pirkkalainen, J.-M. *et al.* Cavity optomechanics mediated by a quantum two-level system. *Nat. Commun.* 6:6981 doi: 10.1038/ncomms7981 (2015).

## Supplementary Material

Supplementary Information Supplementary Figures 1-9, Supplementary Methods, Supplementary References.
