# Supplementary material for: Cavity optomechanics mediated by a quantum two-level system
Source: Nat Commun. 2015 Apr 27;6:6981. doi: 10.1038/ncomms7981 (PMC4421846; doi:10.1038/ncomms7981)
Supplement: Supplementary Information — Supplementary Figures 1-9, Supplementary Methods, Supplementary References. [file ncomms7981-s1.pdf]

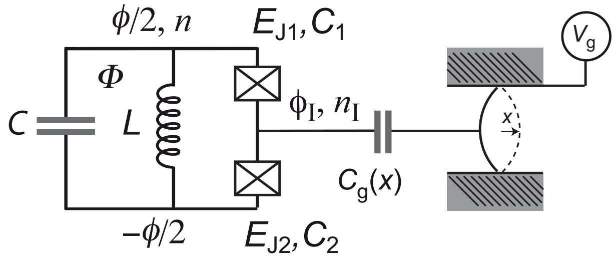

Supplementary Figure 1: **Circuit scheme of the cavity-qubit-mechanics tripartite system**. The flexural micromechanical resonator is shown on the right. It is biased by a constant voltage V_g. Through a movable capacitance, it is connected to a Josephson junction qubit consisting of the two Josephson junctions marked by the crossed boxes. The qubit is coupled to a microwave resonator described by the inductor and capacitor.

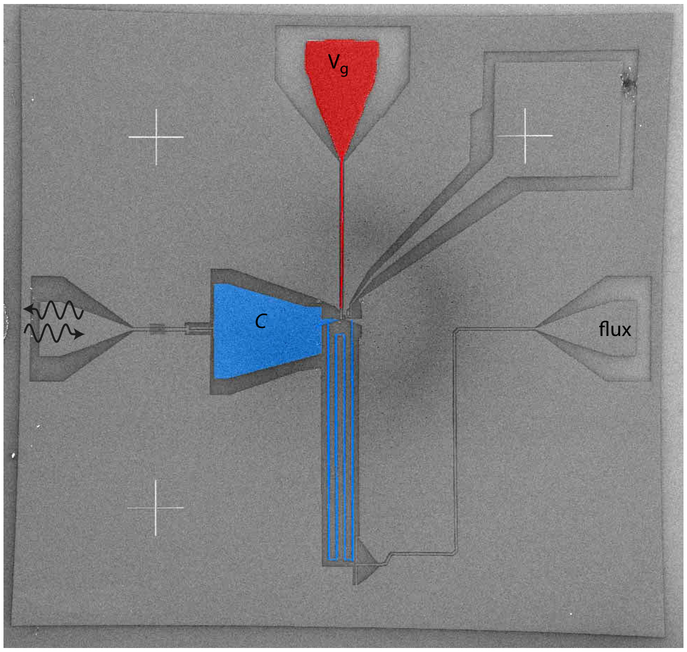

Supplementary Figure 2: **Layout of the chip.** The gray area covering most of the chip is a ground plane. The bonding pad in the upper right corner is for a test junction situated close to the center. The cavity is marked in blue, and the connections to the mechanical resonator in red.

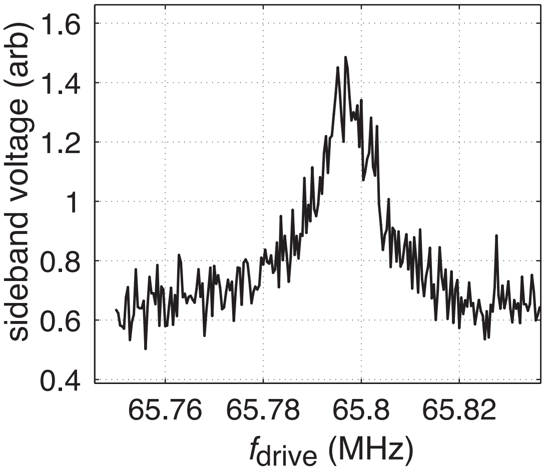

Supplementary Figure 3: **Linear-optomechanical characterization of the mechanical resonator.** The mechanics was actuated with an ac voltage of frequency ω_{drive}. The intrinsic linewidth is obtained as $\gamma_m/2\pi = 15$ kHz, and frequency $\omega_m/2\pi = 65.8$ MHz at $V_g = 1$ V.

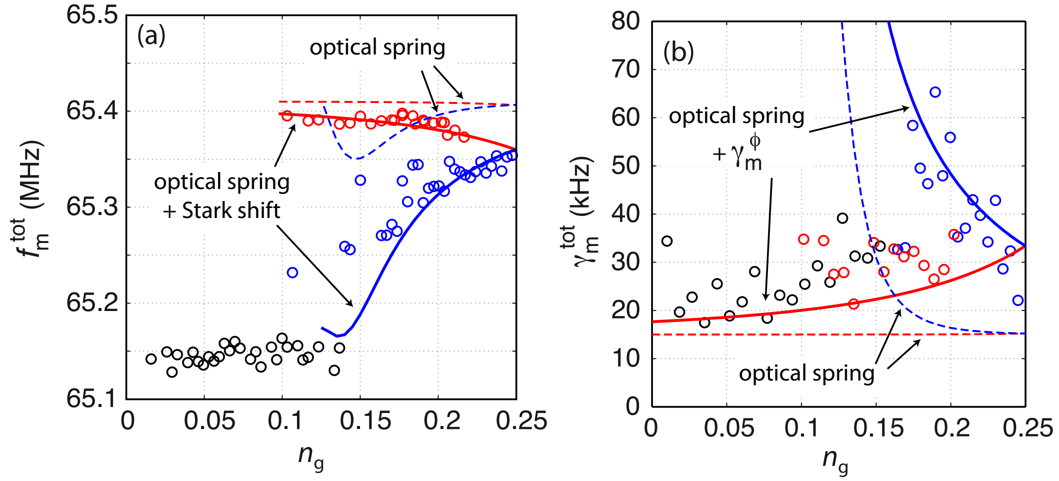

Supplementary Figure 4: **Modeling of the measured effective mechanics**. (a), The solid lines indicate the total effect including the Stark shift, whereas the dashed lines are the contribution due to the optical spring. (b), Similar to (a), except the total effect is partially contributed by the qubit-induced damping. The data are from Figs. 3a and 3b in the main text.

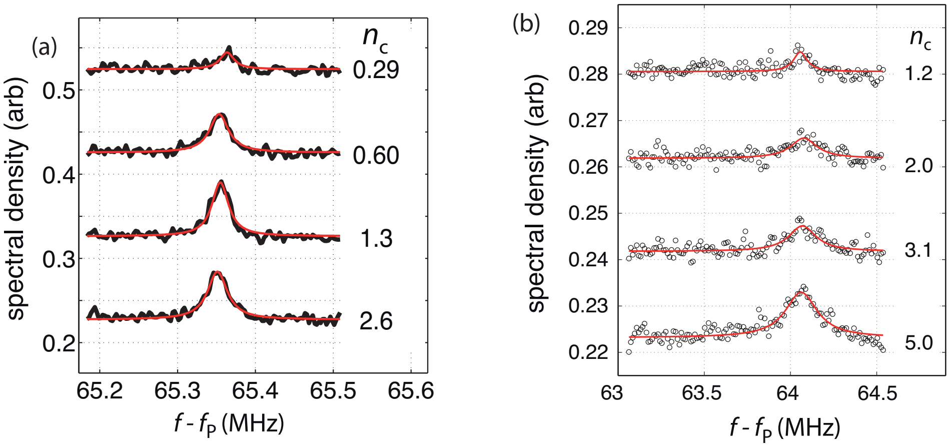

Supplementary Figure 5: **Thermal sidebands**. (a), Thermal motion peaks for different cavity pump photon numbers as marked. $V_g = 6.5$ V, $\Phi/\Phi_0 \simeq 0.39$, $g_0/2\pi \simeq 0.7$ MHz. (b), as panel (a), but higher $V_g = 9.5$ V yielding higher $g_0/2\pi \simeq 1.0$ MHz. Notice the different horizontal as well as vertical scale as compared to (a).

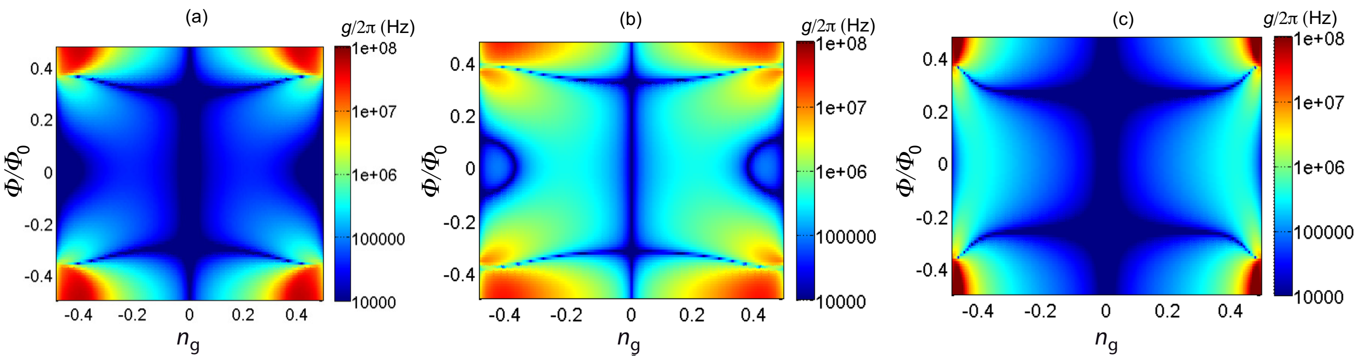

Supplementary Figure 6: **Theoretical predictions for the radiation-pressure coupling**. The plots display the coupling $g/2\pi$ over the all range of the control parameters, namely external flux Φ and the gate charge n_g. The numbers also match the amplification factor g/g_0 if the bare coupling $g_0/2\pi = 1$ Hz which is a typical value (for the present device, $g_0/2\pi \sim 1$ Hz). (a) The present sample. (b) Otherwise as presently, but a high-impedance cavity $C = 0.033$ pF, $L = 33.2$ nH. (c) Otherwise as presently, but the qubit has been tweaked more towards a charge qubit, i.e., $E_J/E_C = 0.5$.

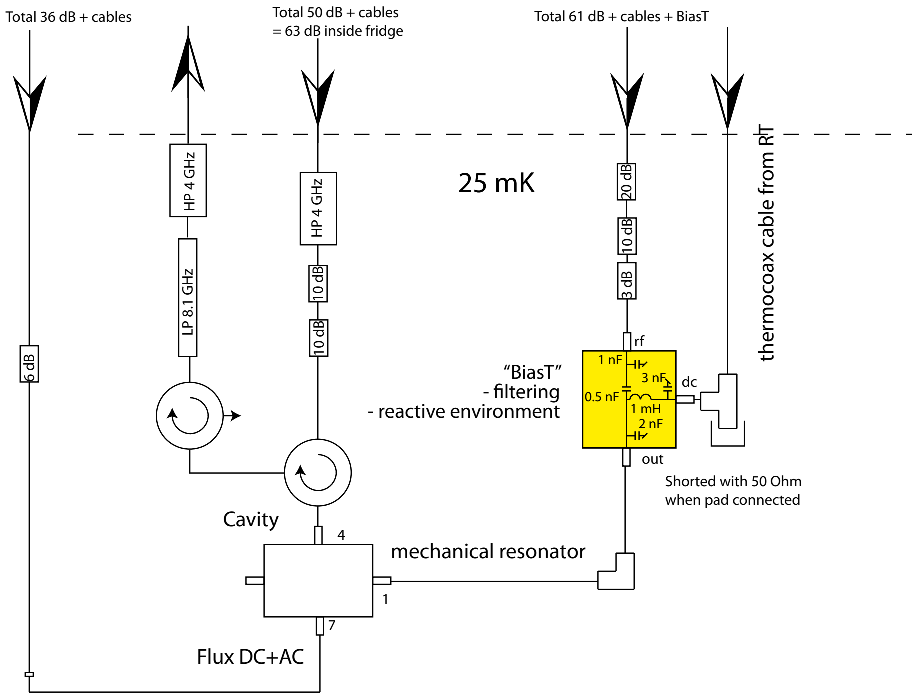

Supplementary Figure 7: **Experimental wiring inside the dilution refrigerator**. We carry out one-port measurements from the device mounted inside a sample box shown by the white rectangle. Two circulators isolate the device from the low-noise amplifier at 4 Kelvin stage of the cryostat. The thermal emission signal due to the mechanical resonator is further amplified at room temperature, and detected with a spectrum analyzer.

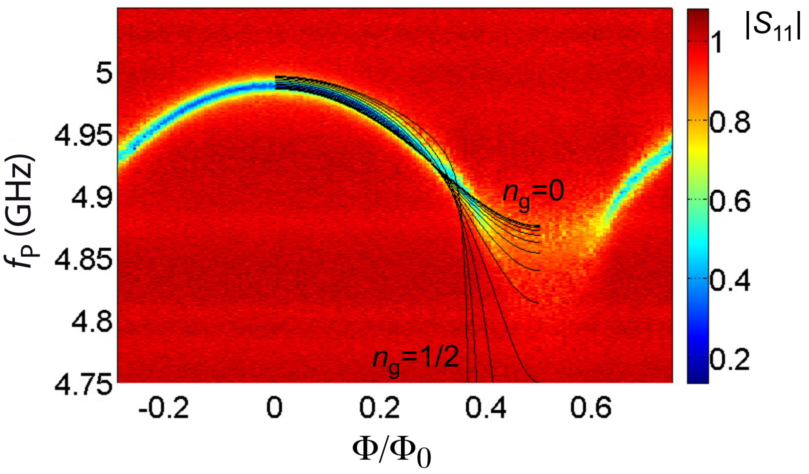

Supplementary Figure 8: **Flux dependence of the cavity response.** The overlaid solid lines are the theoretical prediction from Eq. (10). Each curve is plotted with a given n_g value between 0...1/2 as indicated in the figure.

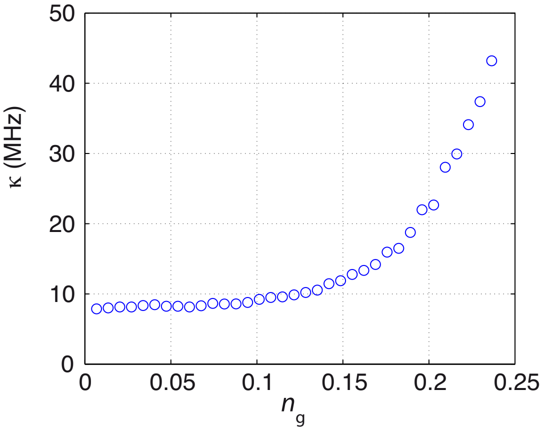

Supplementary Figure 9: **Cavity total linewidth as a function of gate charge of the qubit.** The linewidth broadens due to background charge dephasing.

Supplementary methods

The qubit

Let us first present the charge qubit, marked in green background in Fig. 1c in the main text. The qubit has the junction capacitances C_1, C_2, and the gate capacitance

$$C_g(x) = C_{g0} + \delta C_g(x)\,, \tag{1}$$

which has a little moving part $\delta C_g(x)$ depending on the displacement x of the mechanical resonator. The gate charge is defined as

$$n_g(x) = \frac{V_g C_g(x)}{2e}\,. \tag{2}$$

It is the important control parameter setting the energy difference of having zero or one Cooper pairs on the island. Without the explicit x-argument, we mean the static offset value of the gate charge

$$n_g = \frac{V_g C_{g0}}{2e}\,. \tag{3}$$

Expanding $\delta C_g(x)$ to first order in displacement, we obtain the motional contribution to the gate charge:

$$\delta n_g = \frac{V_g}{2e}\left(\frac{\partial C_g}{\partial x}\right) x\,. \tag{4}$$

The first-order expansion is valid if $\delta C_g(x) \ll C_{g0}$ which holds well because the typical displacement amplitudes at tens of mK temperatures are of the order 20 fm which is much smaller than the capacitor plate separation ~ 50 nm.

The charging energy of the qubit is

$$E_C = \frac{e^2}{2(C_{g0} + C_1 + C_2)}\,. \tag{5}$$

The qubit has the Hamiltonian

$$H_{\mathrm{QB}} = -\sum_{j=x,y,z} B_j \sigma_j / 2\,. \tag{6}$$

In the full charge qubit limit ($E_J \ll E_C$), we can work in Cooper-pair charge-state restriction $\mathrm{int}(n_g)$ and $\mathrm{int}(n_g)+1$. Then, $B_x = (E_{J1} + E_{J2})\cos(\phi/2)$, $B_y = -(E_{J1} - E_{J2})\sin(\phi/2)$ and $B_z = -4E_C(1 - 2n_g)$ are the effective magnetic fields, and σ_j are Pauli matrices acting on the space spanned by the Cooper-pair charge states $|\mathrm{int}(n_g)\rangle$ and $|\mathrm{int}(n_g)+1\rangle$, and ϕ is the phase difference of the superconducting order parameters across the junction. In the absence of the cavity degree of freedom, the phase would relate to an external magnetic field applied through the superconducting loop as $\phi = 2\pi\Phi/\Phi_0$. The ground state energy is $E_{\mathrm{QB}} = -\sqrt{\sum_j B_j^2}/2 \equiv -B/2$. The charge-qubit analytical results hold well (except around $n_g = 0$) up to roughly $E_J/E_C \lesssim 5$ at the current flux bias regime which is around half flux quantum, that is, also for the present experiment.

Full system

Next we will discuss the full tripartite system which is represented by the lumped electromagnetic element model, as shown in Supplementary Figure 1. A magnetic flux Φ is externally applied through the superconducting loop. This loop also forms the cavity inductance.

The x-dependence of gate charge gives the coupling energy of the qubit and the mechanical resonator as

$$g_m = \frac{4E_C V_g}{e}\left(\frac{\partial C_g}{\partial x}\right) x_{\mathrm{zp}}\,. \tag{7}$$

For deriving the Hamiltonian, we use for the cavity node the phase ϕ and the Cooper-pair number n, respectively as the canonical coordinate and momenta. Similarly, ϕ_I and n_I are those for the qubit island.

We define the charging energy of the cavity:

$$E_{Cc} = \frac{e^2}{2C}\,, \tag{8}$$

which in a typical case satisfies $E_{Cc} \ll E_C$.

An initial form for the Hamiltonian is obtained using standard techniques as

$$\begin{aligned} H = 4E_{Cc}n^2 + 4E_C\left(n_I - n_g(x)\right)^2 + \frac{\hbar^2}{4e^2}\frac{(\phi - 2e\Phi/\hbar)^2}{2L} + \\ - E_{J1}\cos(\phi/2 - \phi_I) - E_{J2}\cos(\phi/2 + \phi_I) + \frac{1}{2}m\omega_m^0 x^2 + \frac{p^2}{2m}\,. \end{aligned} \tag{9}$$

The last two terms contain the potential and kinetic energies of the free mechanical resonator. Equation (9) is then quantized with the operators for the cavity $(a^\dagger, a)$ and mechanics $(b^\dagger, b)$, obtaining

$$\begin{aligned} H = \hbar\omega_c^0 a^\dagger a + \hbar\omega_m^0 b^\dagger b + 4E_C(n_I - n_g(x))^2 + \\ - (E_{J1} + E_{J2})\cos\left[\eta(a^\dagger + a) + \pi\Phi/\Phi_0\right]\cos\phi_I - (E_{J1} - E_{J2})\sin\left[\eta(a^\dagger + a) + \pi\Phi/\Phi_0\right]\sin\phi_I\,. \end{aligned} \tag{10}$$

Here, $\eta = \sqrt{e^2\sqrt{L/C}/(2\hbar)}$ is the Lamb-Dicke parameter for the cavity-qubit coupling. It has the same role as the corresponding parameter in trapped ion studies. If $\eta \ll 1$, the cavity-qubit interaction can be linearized as for a trapped ion in the Lamb-Dicke regime.

In order to illustrate a simple result at this point, we further write the qubit operators in two charge-state restriction as in Eq. (6). The condition when this is rigorously valid, from the point of view of the qubit-mechanics coupling, is that the motional gate charge in Eq. (4) is much smaller than one, for then the charge fluctuations due to the vibrations are small. The latter condition is well satisfied in experiment. We obtain that at the maximum coupling ($V_g \sim 10$ V), the zero-point motion corresponds to $\delta n_g \sim 5 \times 10^{-3}$, and the thermal motion is only a couple of times bigger. We use the standard Pauli matrices σ_i: $\sin\phi_I = -\sigma_y/2$, $\cos\phi_I = \sigma_x/2$. We also suppose $E_{J1} = E_{J2} = E_J/2$. Equation (10) then simplifies to

$$H = \hbar\omega_c^0 a^\dagger a + \hbar\omega_m^0 b^\dagger b + 2E_C(1 - 2n_g)\sigma_z - \frac{E_J}{2}\cos(\pi\Phi/\Phi_0)\sigma_x - g_m(b^\dagger + b)\sigma_z + \frac{E_J}{2}\eta(a^\dagger + a)\sin(\pi\Phi/\Phi_0)\sigma_x\,. \tag{11}$$

The last term in Eq. (11) is the linear qubit-cavity interaction, of a typical form with the coupling energy $g_c \sim \eta E_J/2$. Depending on the value of the Lamb-Dicke parameter η which can become of the order of unity, the qubit-cavity coupling can be as high as the bare cavity frequency. In this case, the system would be beyond even the ultra-strong coupling regime of (circuit) QED. In the present work, we have $g_c/\omega_c \sim 10$ %, so we are somewhat in the ultra-strong regime. Regarding the Lamb-Dicke regime, we have $\eta \simeq 0.1$, and hence the Lamb-Dicke limit holds when the cavity is in the vacuum state. However, the pump microwave will excite $a, a^\dagger$ such that the driven system soon escapes out from the Lamb-Dicke regime when $a^\dagger a \sim 1$.

One can now get an intuitive picture of the effective cavity optomechanical system. In the full charge qubit limit, $E_J \ll E_C$, the mechanics coupling is longitudinal and shifts the qubit energy by the amount

$$B \simeq B_z \Longrightarrow B_z(x) = B_z + 2g_m(b^\dagger + b)\,. \tag{12}$$

The cavity then exhibits its own ac Stark shift due to the presence of the qubit. We define the qubit-cavity detuning $\Delta = B - \omega_c^0$. The shifted cavity frequency now is

$$\omega_c^0 \Longrightarrow \omega_c^0 - \frac{g_c^2}{\Delta(x)} = \omega_c^0 - \frac{g_c^2}{\Delta + 2g_m(b^\dagger + b)} = \omega_c^0 - \frac{g_c^2}{\Delta} + \frac{2g_m g_c^2}{\Delta^2}(b^\dagger + b)\,. \tag{13}$$

The last term is the radiation-pressure coupling.

A more rigorous way of derivation which also sheds light on the validity of approximations, is as follows.

With a view to diagonalizing H with respect to the qubit degrees of freedom, Eq. (11) can be written as

$$H = \hbar\omega_c^0 a^\dagger a + \hbar\omega_m^0 b^\dagger b - \frac{B_3}{2}\sigma_z - \frac{B_1}{2}\sigma_x \tag{14}$$

with $B_3 = -4E_C(1 - 2n_g) + 2g_m(b^\dagger + b)$, and $B_1 = E_J\left[\cos(\pi\Phi/\Phi_0) - \eta(a^\dagger + a)\sin(\pi\Phi/\Phi_0)\right]$. The new variables B_1 and B_3 closely resemble B_x and B_z, respectively, in Eq. (6). Within the usual AC-Stark effect picture, invoking RWA for the term σ_x, H can be written as

$$H = \hbar\omega_c^0\left(a^\dagger a - \frac{\sigma_z + 1}{2}\right) + \hbar\omega_m^0 b^\dagger b - \frac{\hbar\Delta}{2}\sigma_z - \frac{B_-}{2}\sigma_+ - \frac{B_+}{2}\sigma_- \tag{15}$$

with $\Delta = B_3 - \omega_c^0$, and $B_- = E_J\left[\cos(\pi\Phi/\Phi_0) - \eta\sin(\pi\Phi/\Phi_0)\right]a$, and $B_+ = B_-^\dagger$.

It is possible to apply a unitary (Schrieffer-Wolff) transformation effectively diagonalizing H with respect to σ_i, while treating a, $a^\dagger$, b and $b^\dagger$ as c-numbers – for an extensive discussion of the Schrieffer-Wolff transformation see e.g. Ref. [1]. For convenience we define $B_3^0 = -4E_C(1-2n_g)$, $\Delta^0 = B_3^0 - \omega_c^0$, $B_1^0 = E_J\cos(\pi\Phi/\Phi_0)$, $B^0 = \left[\left(B_1^0\right)^2 + \left(B_3^0\right)^2\right]^{1/2}$ and $g_c = E_J/2\sin(\pi\Phi/\Phi_0)\,\eta$. The projection of H on the qubit ground state subspace is given by

$$H_{q0} = \hbar\omega_c^0 a^\dagger a + \hbar\omega_m^0 b^\dagger b - \sqrt{\left[\Delta^0/2 + g_m(b^\dagger + b)\right]^2 + (B_1^0/2 - g_c a^\dagger)(B_1^0/2 - g_c a)}, \tag{16}$$

Assuming that $g_m/|B^0|\langle b^\dagger + b\rangle$, $g_c/|B^0|\langle a^\dagger + a\rangle \ll 1$ we can consider the non-diagonal cavity and the mechanics dynamics as a perturbation with respect to the unperturbed Hamiltonian

$$H_{q0}^0 = \hbar\omega_c^0 a^\dagger a + \hbar\omega_m^0 b^\dagger b - \frac{|B^0|}{2}. \tag{17}$$

In order to explicitly outline the validity of the perturbative expansion leading to the effective radiation-pressure interaction, we write H_{q0} as

$$H_{q0} = H_{q0}^0 + \frac{|B^0|}{2}\left\{1 - \sqrt{1 - \left\{B_1^0/2\,g_c(a^\dagger + a) - \Delta^0 g_m(b^\dagger + b) - \left[g_m^2(b^\dagger + b)^2 + g_c^2 a^\dagger a\right]\right\}/|B^0|^2}\right\}. \tag{18}$$

The radiation-pressure coupling between the effective cavity and the effective mechanics is then obtained by expanding Eq. (18) up to second order. Notice that the qubit-mechanics coupling need not to be compared to the mechanical frequency in order for the expansion to be valid. This is relevant in the present experiment since g_m reaches values more than twice ω_m (or ω_m^0). The conditions for the relevant expansion parameters written after Eq. (16) are well satisfied in the experiment.

The theoretical results for the energy levels used in Supplementary Figure 8, and in the main text in Figs. 2c, 3 a,b are obtained by numerical diagonalization of the "full" model in Eq. (10). Notice that for the numerics, we do not need to be in the charge qubit limit, but instead we can have basically an arbitrary E_J/E_C ratio. Similarly, the numerical approach goes beyond the linearized Lamb-Dicke limit of Eq. (11).

Let us next discuss in more detail how the mechanical resonator is affected in the tripartite system. Based on Eq. (11) one can see that exciting the qubit to the excited state corresponds to a shift of $g_m/(\hbar\omega_m)$ of the equilibrium position of the mechanical resonator. In practice this corresponds to some tens of fm. We assume that such a small constant shift does not change the elastic parameters, in particular, the bare resonator frequency. On the other hand, a time-dependent shift of the position will yield the optical spring effect and Stark effect measured in the experiment. The effective mechanical frequencies ω_m^{tot} arise as follows. The starting point is the "bare" frequency ω_m. Due to the interaction with the *qubit* the mechanical resonator will exhibit an ac Stark shift, which is obtained by expanding Eq. (18) to second order in $\Delta^0/B^0(b^\dagger + b)$:

$$\omega_m = \omega_m^0 - \frac{2g_m^2 B_x^2}{B^3}. \tag{19}$$

As discussed in the main text, the measured mechanical frequency is then the sum of ω_m and the contribution by the optical spring, viz. $\omega_m^{\mathrm{tot}} = \omega_m + \delta\omega_m^{\mathrm{opt}}$. One can separate the two contributions by studying the prediction for their effects. The separation is possible because the optical spring effect depends on the pumping, but the Stark effect does not. We plot the data in Fig. 3 but overlaid with these two contributions, in Supplementary Figure 4. In fact, as seen in the plot, with typical parameters, ω_m^{tot} is dominated by the Stark shift.

Next we discuss briefly the prospects of further increasing the radiation-pressure coupling. The boosted coupling is

$$g = x_{\mathrm{zp}}\frac{\partial\omega_c}{\partial x} = x_{\mathrm{zp}}\frac{V_g}{2e}\frac{\partial\omega_c}{\partial n_g}\frac{\partial C_g}{\partial x}. \tag{20}$$

The term $\frac{\partial\omega_c}{\partial n_g}$ is numerically obtained from Eq. (10). In Supplementary Figure 6we display some theoretical predictions for the present device over the full range of the control parameters Φ and n_g, as well as for two hypothetical devices. We see that for the present sample, the couplings can in principle become as high as several tens of MHz. However, these values occur in the range $n_g > 0.35$ where the gate charge fluctuations presumably dephase the cavity so that the cavity resonance is not visible any more (see Fig. 2c in main text). Hence, with the technology at hand, operating in this regime is not feasible, but in principle this is not a fundamental setback nevertheless. At the moment we see as the most promising approach to choose a high-impedance cavity (Supplementary Figure 6c), where the capacitance has been minimized and hence the movable capacitance has a bigger role.

Supplementary References

[1] Bravyi, S., DiVincenzo, D. P. & Loss, D. Schrieffer-Wolff transformation for quantum many-body systems. *Ann. Phys.-New York* **326**, 2793–2826 (2011).
